# CAR: contig assembly of prokaryotic draft genomes using rearrangements

**DOI:** 10.1186/s12859-014-0381-3

**Published:** 2014-11-28

**Authors:** Chin Lung Lu, Kun-Tze Chen, Shih-Yuan Huang, Hsien-Tai Chiu

**Affiliations:** Department of Computer Science, National Tsing Hua University, Hsinchu, 300 Taiwan; Department of Chemistry, National Cheng Kung University, Tainan City, 701 Taiwan

**Keywords:** Bioinformatics, Contig assembly, Rearrangement

## Abstract

**Background:**

Next generation sequencing technology has allowed efficient production of draft genomes for many organisms of interest. However, most draft genomes are just collections of independent contigs, whose relative positions and orientations along the genome being sequenced are unknown. Although several tools have been developed to order and orient the contigs of draft genomes, more accurate tools are still needed.

**Results:**

In this study, we present a novel reference-based contig assembly (or scaffolding) tool, named as CAR, that can efficiently and more accurately order and orient the contigs of a prokaryotic draft genome based on a reference genome of a related organism. Given a set of contigs in multi-FASTA format and a reference genome in FASTA format, CAR can output a list of scaffolds, each of which is a set of ordered and oriented contigs. For validation, we have tested CAR on a real dataset composed of several prokaryotic genomes and also compared its performance with several other reference-based contig assembly tools. Consequently, our experimental results have shown that CAR indeed performs better than all these other reference-based contig assembly tools in terms of sensitivity, precision and genome coverage.

**Conclusions:**

CAR serves as an efficient tool that can more accurately order and orient the contigs of a prokaryotic draft genome based on a reference genome. The web server of CAR is freely available at http://genome.cs.nthu.edu.tw/CAR/ and its stand-alone program can also be downloaded from the same website.

**Electronic supplementary material:**

The online version of this article (doi:10.1186/s12859-014-0381-3) contains supplementary material, which is available to authorized users.

## Background

The draft genomes produced by most assemblers for next generation sequencing (NGS) are just collections of independent contigs, whose relative positions and orientations along the genome being sequenced are unknown. To address this problem, a process called *scaffolding* is then used to order and orient these contigs of a draft genome. An accurate scaffolding is critical and helpful for accomplishing the subsequent *finishing* process, which applies the primer walking technique to closing the gaps between ordered and oriented contigs. Currently, many NGS assemblers utilize the information of paired-end reads (or mate-pair reads) to produce the scaffolds, each of which is a set of ordered and oriented contigs [1-3]. Such paired-end reads can be generated by sequencing both ends of large DNA molecules like bacterial artificial chromosomes (BAC), thus producing pairs of sequenced reads with known relative orientation and approximate distance. As a result, if the two paired-end reads can be mapped to two individual contigs unambiguously, the relative order and the distance between these two contigs can thus be correctly identified. In practice, a mixture of paired-end reads with various distances is needed to improve the accuracy of the scaffolding by reducing the experimental errors. In computation, such a scaffolding process can be modeled as a combinatorial optimization problem, which aims to order and orient the input contigs in a manner that maximizes the number of supporting paired-end reads. Unfortunately, this problem is computationally difficult, because it has been shown to be NP-hard [4], meaning that finding an efficient polynomial time algorithm to solve this problem is highly unlikely. An alternative approach to order and orient the contigs of a draft genome is to take advantage of and utilize the finished genome of a related organism as a reference [5]. In principle, the contigs of a draft genome can be mapped to a reference genome and their positions on the reference genome are then used to infer the scaffolding of contigs. Thus far, several tools using this approach have been developed, such as Projector 2 [6], OSLay [7], ABACAS [8], Mauve Aligner [9], fillScaffolds [10], r2cat [11], CONTIGuator [12] and SIS [13].

In this study, we present a novel reference-based contig assembly (or scaffolding) tool named as CAR (short for “Contig Assembly using Rearrangements”) that can efficiently and more accurately order and orient the contigs of a prokaryotic draft genome based on a reference genome of a related organism. The kernel program of CAR was implemented using a different but more accurate algorithm we recently developed [14]. In principle, we formulated the reference-based scaffolding problem as the following combinatorial optimization problem: Given a set of contigs for a draft genome and a reference genome, the goal of the problem is to order and orient the contigs of the draft genome in a way that minimizes the rearrangement distance between the assembled draft genome and the reference genome. The rationale of defining such a reference-based scaffolding problem is as follows. Firstly, the draft and reference genomes in this problem are represented by signed permutations of *n* integers, where each integer represents a conserved genetic marker (gene or synteny block) shared between the draft and reference genomes and its associated sign indicates the strandedness of the corresponding genetic marker. If the draft and reference genomes are phylogenetically closely related, then the contig assembly of the draft genome may have a genetic-marker order similar to that of the reference genome, since the global (or large-scale) mutations of genome rearrangements between them are relatively rare [15]. Note that the reference-based scaffolding problem we formulated above is a variant of the one defined by Gaul and Blanchette [16], because the reference genome used by Gaul and Blanchette can be a draft genome (but not necessarily a finished genome as required here). As already shown in our previous study [14], we used the permutation groups to design an efficient algorithm to solve this reference-based scaffolding problem, where the rearrangement distance in the problem was measured by reversals and block-interchanges (also called generalized transpositions) with the weight ratio 1:2 [14]. Reversal and block-interchange are two different kinds of genome rearrangements that can affect the genomic organization of DNA molecules [15]. Reversal affects a segment on a chromosome by reversing this segment as well as exchanging its strands, while block-interchange is a generalized transposition that exchanges two nonoverlapping (but not necessarily adjacent) segments on a chromosome. Usually, transpositions, as well as block-interchanges, occur less frequently than reversals in many evolutionary scenarios. As also discussed in our previous studies [17,18], it is biologically meaningful to assign twice the weight to block-interchanges than to reversals based on the observation of real biological data [19] and the result of computer simulations [20]. It is worth mentioning here that the contigs of a draft genome can be ordered and oriented by our algorithm in $\mathcal {O}(n)$ time [14], where *n* is the number of genetic markers.

CAR is an easy-to-use tool for contig assembly of a prokaryotic draft genome. Given a set of contigs in multi-FASTA format and a reference genome in FASTA format, it can output a list of *scaffolds*, each consisting of the ordered and oriented contigs. To validate CAR, we have tested it on a real dataset composed of several prokaryotic genomes and also compared its performance with several other reference-based contig assembly tools. As a consequence, our experimental results have shown that CAR indeed performs better than all these other reference-based tools in terms of sensitivity, precision and genome coverage.

## Implementation

### Overview

The method we used to implement CAR is described as follows. Note that the genomes considered below are unichromosomal. For the calculation of rearrangement distance, the input draft genome *π* and the reference genome *σ* of our algorithm must be represented as two signed permutations of *n* integers between 1 and *n*, where each integer represents a conserved genetic marker between the draft and reference genomes and its associated sign indicates the strandedness of the corresponding genetic marker. For this purpose, we first used MUMmer’s programs [21], NUCmer and PROmer, with default settings to detect the conserved genetic markers between the draft and reference genomes, where NUCmer is performed on the input nucleotide sequences and PROmer on the amino acid sequences translated from the input nucleotide sequences in all six reading frames. The delta-filter utility program of MUMmer with parameter ‘-1’ was then used to remove the repeated genetic markers from the draft and reference genomes. Subsequently, we applied our algorithm [14] on the obtained signed permutations to order and orient the contigs of the draft genome *π* based on the reference genome *σ*.

### Basic idea of algorithm

The algorithm we designed in [14] was based on permutation groups in algebra, which have been proven to be very useful in the studies of genome rearrangements [17,18]. Basically, we consider the assembly (scaffolding, i.e., ordering and orienting) of two contigs as a rearrangement, called *fusion*, that joins these two contigs into one. Assume that there are *m* contigs in the draft genome *π*. The main job of our algorithm is then to find *m*−1 fusions to join the *m* contigs in *π* such that the rearrangement distance between the resulting contig assembly of *π* and the reference genome *σ* is minimized. For proper modeling of the contigs using permutation groups, we initially add two caps (i.e., dummy genetic markers) to the ends of each contig of *π* and *σ*, resulting in the capped draft genome $\hat {\pi }$ and the capped reference genome $\hat {\sigma }$. We then show that the fusion of two contigs in *π* can be mimicked by a special translocation acting on the corresponding contigs in $\hat {\pi }$, where the *translocation* is a kind of rearrangement that acts on two chromosomes by exchanging their end fragments. Next, we calculate the production of $\hat {\sigma }$ and the inverse of $\hat {\pi }$, from which we can further derive *m*−1 special translocations to act on $\hat {\pi }$ such that their rearrangement effects on original *π* are *m*−1 fusions. In particular, we show that these *m*−1 fusions can be used to optimally join the *m* contigs of *π*, and the whole process of this contig assembly can be finished in linear time. For full details on this algorithm, we refer the reader to our original paper [14].

### Usage of CAR

**Figure 1:**
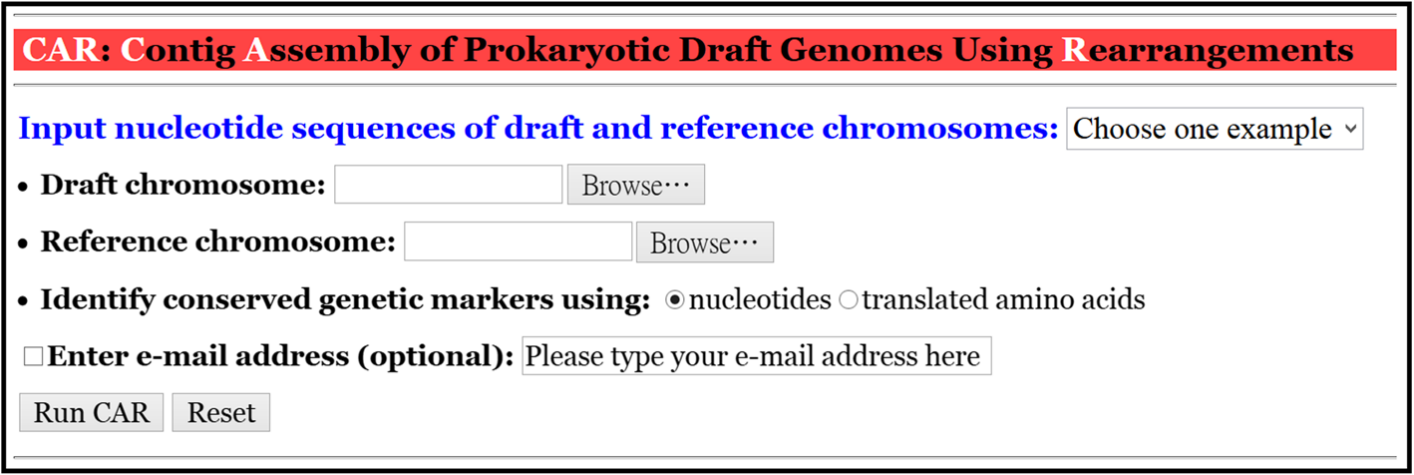
**The web interface of CAR.**

**Figure 2:**
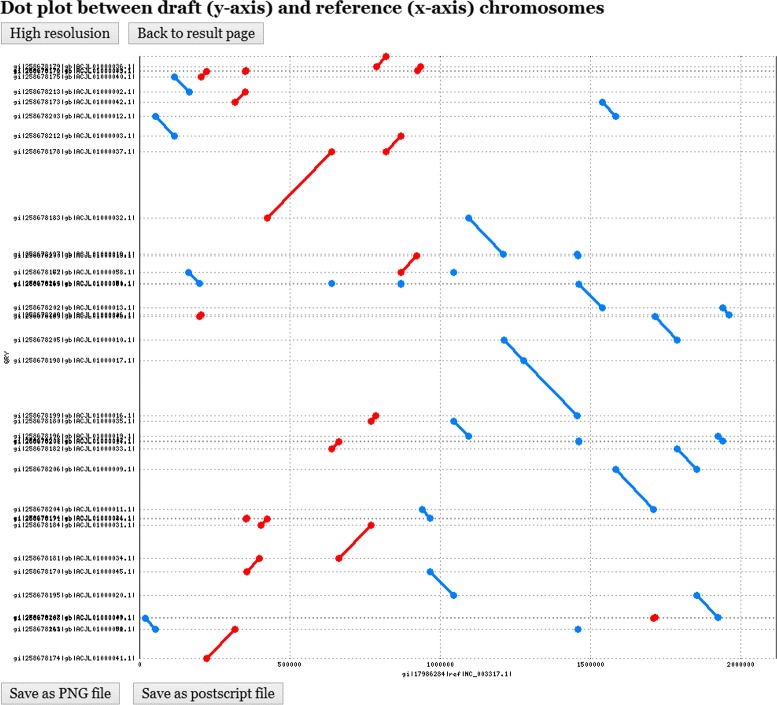
**The dot plot of draft and reference chromosomes before contig assembly.**

**Figure 3:**
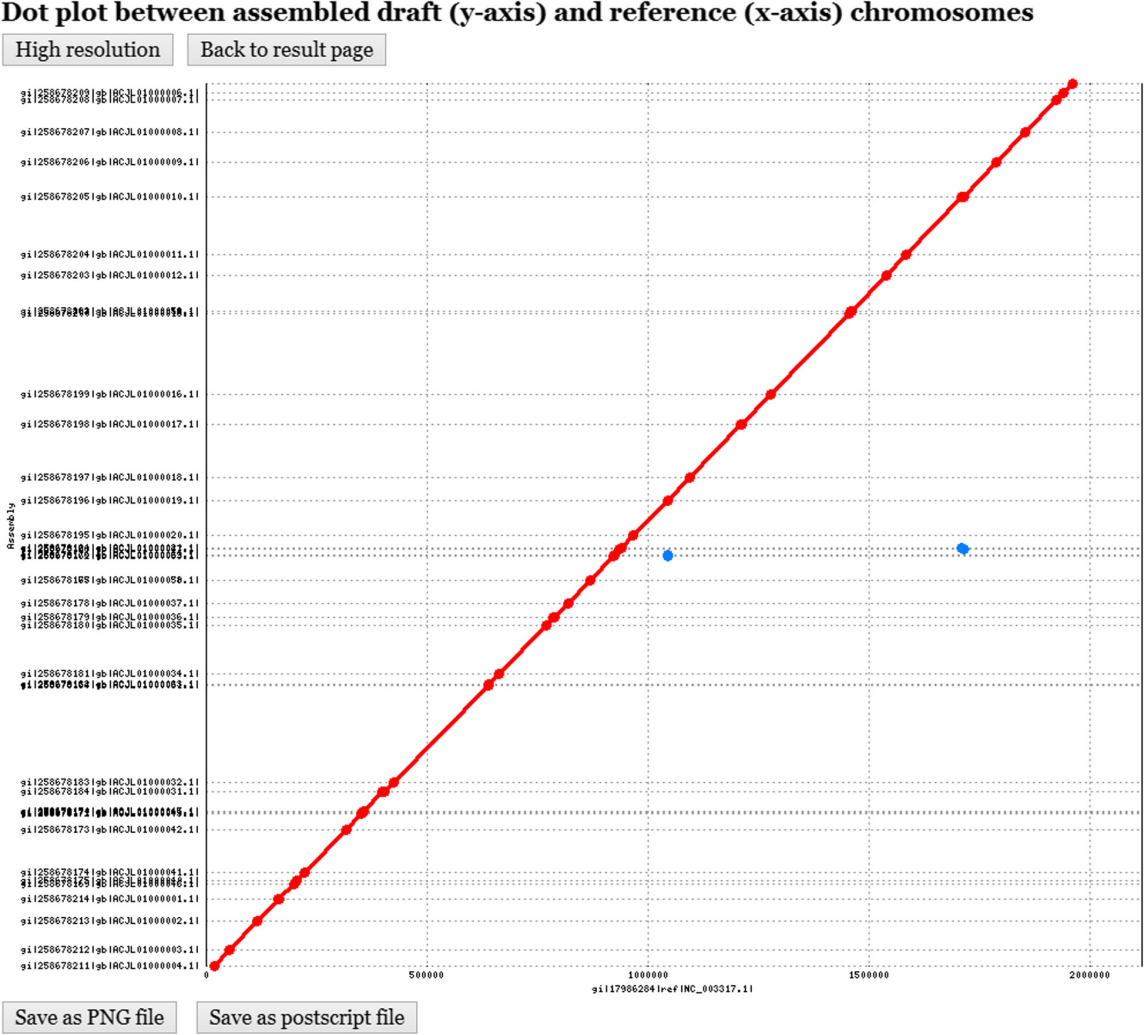
**The dot plot of assembled draft and reference chromosomes after contig assembly.**

The kernel programs of CAR and its web interface were implemented in PHP. Its server is installed on IBM PC with 2.8 GHz processor and 3 GB RAM under Linux system. CAR takes as input a set of contigs of a prokaryotic draft chromosome in multi-FASTA format and a reference chromosome in FASTA format (see Figure 1). Next, CAR automatically identifies conserved genetic markers between the input draft and reference chromosomes either based on their nucleotides or translated amino acids, which can be specified by the user. The user can also choose to run CAR in a batch mode. Subsequently, CAR returns with a contig assembly result of the draft chromosome in a feasible time. Note that for the size of prokaryotic chromosomes, CAR can finish its contig assembly job in several seconds to a couple of minutes. In the output page, CAR first shows the nucleotide sequences of the input draft and reference chromosomes, a dot plot graph between them before performing contig assembly (see Figure 2 for an example), and a user-specified parameter of identifying conserved genetic markers. Note that in the dot-plot graph, the contigs of the draft chromosome are plotted on the *y*-axis, whereas the sequence of the reference chromosome is plotted on the *x*-axis. Moreover, the forward matches are displayed in red and the reverse matches in blue. Next, CAR shows a contig assembly result of the draft chromosome based on the reference chromosome, including total running time, a set of scaffolds and its corresponding multi-FASTA file, a dot plot graph between the assembled draft and reference chromosomes (see Figure 3 for an example), and a comparison of dot-plot graphs between before and after contig assemblies. For more details on using CAR, please refer to the help page of CAR at http://genome.cs.nthu.edu.tw/CAR/help.html.

## Results and discussion

### Testing dataset

For validation, we used a real dataset composed of several prokaryotic genomes to test CAR and compared its performance to eight other reference-based tools of contig assembly (scaffolding), namely Projector2 [6], OSLay [7], ABACAS [8], Mauve Aligner [9], fillScaffolds [10], r2cat [11], CONTIGuator [12] and SIS [13]. This real dataset was used in the study of SIS by Dias *et al*. [13], which contains 19 draft genomes of phylogenetically diverse prokaryotes downloaded from the GenBank of NCBI. Among these 19 prokaryotic genomes, four of them have two chromosomes and the others have only one, thus giving a total of 23 chromosomes in this dataset (as listed in Table 1). Each of these 23 chromosomes was then processed separately by each contig assembly tool. For the draft of each query chromosome, we used 20 closest genomes (excluding the query genome itself) to serve as different reference genomes, which were selected by Dias *et al*. [13] from complete prokaryotic genomes deposited in the GenBank of NCBI according to their phylogenetic distances from the query genome. The purpose of choosing the 20 closest other genomes instead of only the closest one is to understand how the accuracy performance of a contig assembly tool changes accordingly with different evolutionary distances between query and possible reference genomes.

**Table 1:** **Draft chromosomal genomes used in the testing dataset**

**Organism**	**Accession no.**	**Size (bp)**	**# Contig**	**COV (%)**
*Aciduliprofundum boonei* T469	NC_013926	1,486,778	35	98.63
*Bacillus subtilis* 168	NC_000964	4,215,606	5	99.97
*Bifidobacterium longum* DJO 10A	NC_010816	2,375,792	58	85.47
*Brucella melitensis* bv 1 16M (I)	NC_003317	2,117,144	41	90.83
*Brucella melitensis* bv 1 16M (II)	NC_003318	1,177,787	12	99.77
*Brucella pinnipedialis* B2 94 (I)	NC_015857	2,138,342	55	87.47
*Brucella pinnipedialis* B2 94 (II)	NC_015858	1,260,926	34	84.38
*Burkholderia thailandensis* E264 (II)	NC_007650	2,914,771	15	70.34
*Burkholderia thailandensis* E264 (I)	NC_007651	3,809,201	28	89.90
*Chlamydia muridarum* Nigg	NC_002620	1,072,950	4	99.09
*Clostridium cellulovorans* 743B	NC_014393	5,262,222	297	96.54
*Corynebacterium aurimucosum* ATCC 700975	NC_012590	2,790,189	90	92.94
*Corynebacterium efficiens* YS 314	NC_004369	3,147,090	118	95.09
*Micrococcus luteus* NCTC 2665	NC_012803	2,501,097	126	86.25
*Mycobacterium tuberculosis* H37Ra	NC_009525	4,419,977	220	76.84
*Mycoplasma genitalium* G37	NC_000908	580,076	24	78.54
*Saccharopolyspora erythraea* NRRL 2338	NC_009142	8,212,805	238	97.10
*Selenomonas sputigena* ATCC 35185	NC_015437	2,568,361	53	94.01
*Stigmatella aurantiaca* DW 431	NC_014623	10,260,756	472	99.10
*Streptococcus pneumoniae* TIGR4	NC_003028	2,160,842	209	90.31
*Vibrio* Ex25 (I)	NC_013456	3,259,580	176	91.43
*Vibrio* Ex25 (II)	NC_013457	1,829,445	33	95.31
*Yersinia pestis* Nepal 516	NC_008149	4,534,590	17	83.86

The column “# Contig” shows the number of contigs selected for experiments of contig assembly by excluding, for example, those contigs not mapped to reference chromosome. The column “COV” gives the fraction of each genome or chromosome covered by selected contigs.

### Comparisons on sensitivity and precision

The number of correct contig joins (or adjacency) is the main quality measure for a scaffold [13]. A join of two contigs in a scaffold is said to be *correct* if they are also consecutive in the completely finished query genome. Note that in the above dataset the genomic sequences of the species are completely finished and available from the GenBank of NCBI. Using these completely finished genomes, we can thus derive a *reference order* for the collection of contigs of each draft chromosomal genome to serve as the standard of truth in our evaluation. The reference order was derived by mapping the contigs to their corresponding finished chromosomal genome and placing them on the positions where they gained the most matches. Note that those contigs that were not matched at all were excluded in the reference order. Let *P* denote the number of all contig joins in the reference order. For the output of each contig assembly tool, we compared it with the reference order by counting the number of all contig joins that also occur in the corresponding reference order as *true positive* (denoted by *TP*) and the number of the others as *false positive* (denoted by *FP*). Using these values of each contig assembly tool, we computed the *sensitivity* defined as (*TP*×100)/*P* and the *precision* as (*TP*×100)/(*TP*+*FP*).

Among all the contig assembly tools we tested, ABACAS, fillScaffolds, SIS and CAR can choose either NUCmer or PROmer to identify conserved genetic markers between draft and reference chromosomes. For ABACAS, however, only NUCmer was adopted since in our test no contig assembly results were obtained after executing ABACAS with PROmer for several days. On the other hand, we randomized (shuffled) the input order of contigs for each instance to eliminate potential effect of contig order on experimental results. As a result, Tables 2 and 3 show average sensitivity and precision, respectively, over all instances (i) when using the closest chromosomes as the references, (ii) when using the top 10 closest chromosomes as the references, and (iii) when using the top 20 closest chromosomes as the references. These two tables were sorted in descending order according to the values shown in their third column (i.e., average sensitivity/precision obtained when using the top 10 closest chromosomes as the references). As clearly shown in Tables 2 and 3, upon using PROmer to identify conserved genetic markers, CAR gives the best sensitivity and precision in all three cases as compared to the eight other contig assembly tools.

**Table 2:** **Comparison of average sensitivity for various contig assembly tools**

**Tool**	**Closest reference**	**Top 10**	**Top 20**
CAR (PROmer)	62.71 (67.50)	49.87 (56.25)	37.33 (32.25)
SIS (PROmer)	60.82 (67.50)	48.53 (54.55)	36.14 (30.40)
Mauve Aligner	60.19 (65.22)	46.40 (46.88)	32.86 (22.47)
r2cat	61.64 (78.13)	43.56 (38.52)	30.01 (20.51)
CAR (NUCmer)	57.04 (73.68)	43.38 (39.01)	28.19 (7.41)
SIS (NUCmer)	55.41 (72.73)	42.70 (36.67)	27.56 (6.40)
OSLay	48.38 (62.50)	34.43 (12.90)	21.18 (0.60)
fillScaffolds (NUCmer)	49.04 (56.41)	34.23 (21.83)	21.36 (4.53)
fillScaffolds (PROmer)	45.19 (50.00)	33.18 (25.93)	21.76 (8.75)
CONTIGuator	45.66 (50.00)	31.53 (15.43)	19.29 (0.68)
Projector2	42.58 (40.17)	29.18 (20.49)	18.63 (5.00)
ABACAS	33.42 (28.57)	23.64 (0.38)	13.01 (0.00)

This table is sorted in descending order according to the average values shown in the “Top 10” column, where the values in parentheses are medians.

**Table 3:** **Comparison of average precision for various contig assembly tools**

**Tool**	**Closest reference**	**Top 10**	**Top 20**
CAR (PROmer)	68.50 (73.91)	56.54 (66.04)	43.30 (40.00)
SIS (PROmer)	66.47 (73.91)	54.96 (60.00)	41.84 (38.98)
CAR (NUCmer)	63.71 (81.25)	51.49 (56.25)	35.36 (22.22)
SIS (NUCmer)	61.99 (76.92)	50.54 (56.25)	34.36 (20.99)
OSLay	61.86 (75.00)	49.57 (59.41)	38.00 (33.33)
r2cat	65.59 (79.17)	48.38 (48.61)	34.91 (26.67)
Mauve Aligner	60.19 (65.22)	46.41 (46.88)	32.88 (22.47)
CONTIGuator	58.95 (66.67)	41.83 (42.33)	28.23 (11.11)
Projector2	57.85 (64.29)	41.63 (37.50)	29.04 (20.00)
fillScaffolds (NUCmer)	54.50 (59.26)	40.34 (30.88)	26.57 (12.40)
fillScaffolds (PROmer)	48.79 (51.46)	37.14 (29.15)	24.67 (12.50)
ABACAS	46.88 (50.00)	31.54 (14.29)	20.43 (0.00)

This table is sorted in descending order according to the average values displayed in the “Top 10” column, where the values in parentheses are medians.

**Figure 4:**
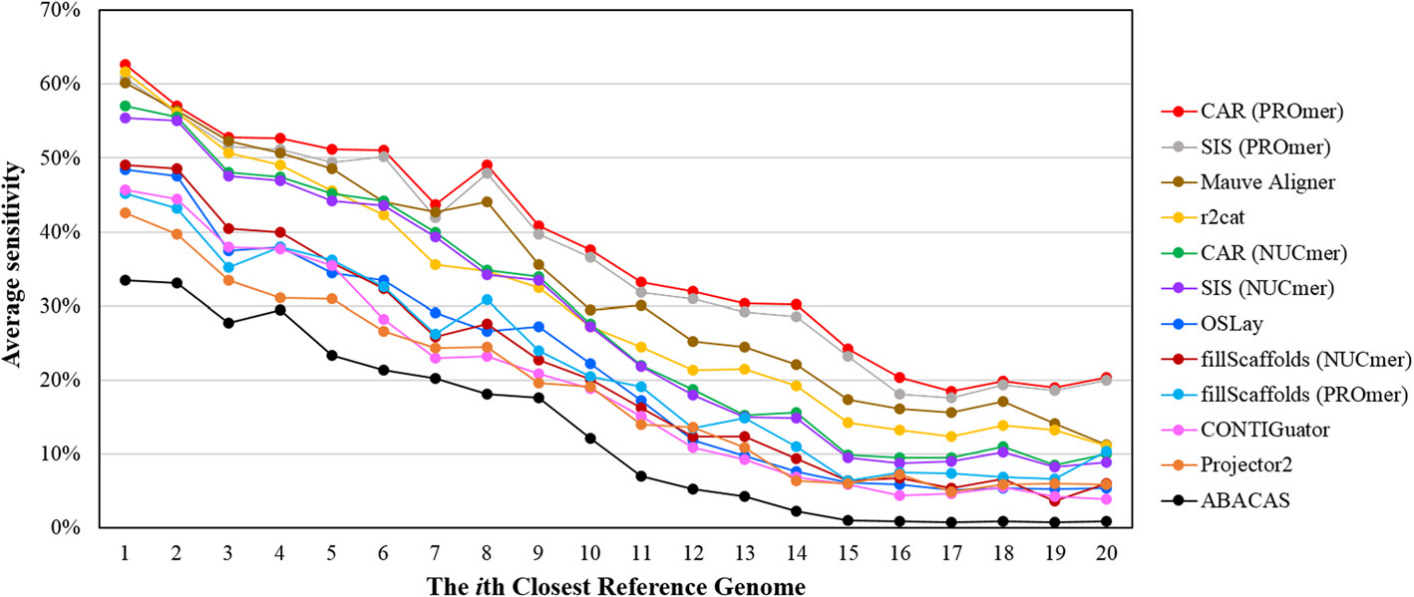
**Average sensitivity obtained by each tool when the reference genome varies from the closest to the farthest in the phylogenetic distance.**

**Figure 5:**
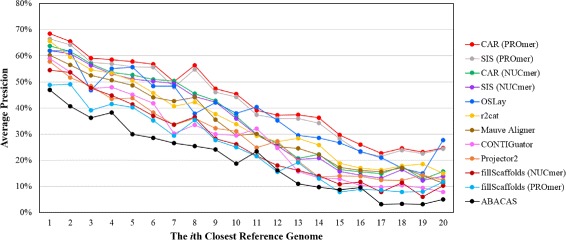
**Average precision obtained by each tool when the reference genome varies from the closest to the farthest in the phylogenetic distance.**

Figures 4 and 5 further show the average sensitivity and precision, respectively, of all contig assembly tools over 23 query chromosomes when the reference genome varies from the closest to the farthest in the phylogenetic distance. Consequently, both their average sensitivity and precision descend along with increasing phylogenetic distance between the query and reference genomes. Nevertheless, CAR (running with PROmer) is still superior to all other contig assembly tools in terms of sensitivity as shown in Figure 4, as well as to almost all of them in terms of precision, except for OSLay when using the 11th or 20th closest genome as the reference, as shown in Figure 5.

Actually, all the contig assembly tools used in this study can be classified into the following two categories: (a) alignment-based tools and (b) rearrangement-based tools. Projector 2 [6], OSLay [7], ABACAS [8], Mauve Aligner [9], r2cat [11] and CONTIGuator [12] belong to the former category of alignment-based tools, while fillScaffolds [10], SIS [13] and CAR belong to the latter category of rearrangement-based tools. The alignment-based tools align contigs or contig ends of a draft genome against a reference sequence, and then ordered and oriented the contigs according to their positions (matches) in the reference. The performance of these tools for ordering and orienting the contigs is highly dependent on the similarity between the draft and reference genomes. If the draft and reference genomes are not similar to a sufficient degree, or their phylogenetic relationship is not very close, the alignment-based tools may place the contigs in an incorrect order. As to the rearrangement-based tools, they attempt to order and orient the contigs by utilizing the comparison of genetic-marker orders between draft and reference genomes. Basically, DNA molecules are subject to local mutations (such as nucleotide substitutions, insertions and deletions) and global mutations (such as genome rearrangements) during their evolution. In contrast to local mutations that normally accumulate rather quickly, genome rearrangements are relatively rare events during evolution, implying that the genetic-marker orders between two species should be more conserved than their nucleotide sequences. This may thus suggest that the rearrangement-based tools should fit better than the alignment-based tools for correctly ordering and orienting the contigs of a draft genome, especially when the draft genome is phylogenetically distant from the reference genome. On the other hand, among the three rearrangement-based tools mentioned above, CAR has better performance when compared to SIS and fillScaffolds. The reason may be as follows. SIS deals with only reversals and searches for inversion signatures to order and orient the contigs in a draft genome. In addition to reversals, fillScaffolds considers other rearrangements, such as transpositions and translocations (including fissions and fusions). It treats each contig as a (linear) chromosome and uses an existing rearrangement algorithm, such as the one proposed by Tesler [22], to order and orient the contigs in a draft genome. However, the purpose of the existing rearrangement algorithm itself is not dedicated to the ordering and orientation of the contigs. CAR herein considers both reversals and block-interchanges (generalized transpositions) and further utilizes an exact algorithm that can optimally solve the reference-based scaffolding problem we formulated in this study. As compared to the exact algorithm used by CAR that can produce mathematically optimal solutions, the algorithms adopted by SIS and fillScaffolds are heuristics that can produce only approximate solutions.

### Comparison on genome coverage

**Figure 6:**
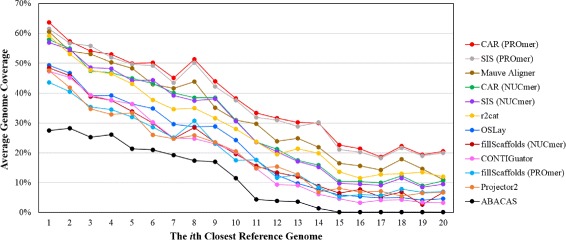
**Average genome coverage obtained by each tool when the reference genome varies from the closest to the farthest in the phylogenetic distance.**

Genome coverage is another quality metric to measure how much of the genome being sequenced is actually covered by the scaffolds generated by a contig assembly tool [13]. We followed the procedure adopted by Dias *et al*. [13] to compute the genome coverage of each contig assembly tool. As mentioned earlier, a contig join that also occurs in the reference order is considered as a correct contig adjacency. For a given contig, if its both ends have correct adjacencies, its whole length is thus counted as contributing to the genome coverage. If only one end of this contig has a correct adjacency, then its half length is counted. If its both ends has no correct adjacencies, this contig is then not considered. Then, the *genome coverage* is defined as the ratio of the sum of contig lengths that are counted according to the aforementioned rules and the sum of all contig lengths. Consequently as shown in Table 4, CAR gives the best genome coverage compared to the eight other contig assembly tools when using PROmer to find conserved genetic markers. As also shown in Figure 6, the average genome coverage of all contig assembly tools over 23 query chromosomes is degraded with increasing phylogenetic distance between the query and reference genomes. However, CAR (running with PROmer) is still better than almost all other tools, except for SIS when using the 3rd and 14th closest genomes as the reference.

**Table 4:** **Comparison of genome coverage for various contig assembly tools**

**Tool**	**Closest reference**	**Top 10**	**Top 20**
CAR (PROmer)	63.73 (74.68)	50.67 (58.82)	37.81 (34.88)
SIS (PROmer)	61.51 (73.85)	49.81 (55.08)	37.00 (33.78)
Mauve Aligner	60.57 (72.30)	46.09 (45.07)	32.54 (22.62)
CAR (NUCmer)	57.87 (76.06)	44.30 (44.23)	29.19 (12.16)
SIS (NUCmer)	56.95 (74.68)	44.21 (47.43)	28.81 (10.60)
r2cat	59.21 (71.69)	41.63 (36.84)	28.85 (19.48)
OSLay	49.36 (68.09)	35.71 (13.85)	21.72 (0.52)
fillScaffolds (NUCmer)	48.47 (61.49)	33.07 (16.28)	20.81 (5.26)
CONTIGuator	47.33 (60.06)	32.87 (17.95)	19.54 (0.44)
fillScaffolds (PROmer)	43.59 (42.91)	31.08 (16.95)	19.99 (7.04)
Projector2	47.54 (51.58)	31.07 (20.10)	20.06 (7.09)
ABACAS	27.48 (8.15)	21.43 (0.12)	11.41 (0.00)

This table is sorted in descending order according to the average values shown in the “Top 10” column, where the values in parentheses are medians.

### Additional results

Additional performance results of all contig assembly tools on individual query chromosomes can be found in Additional file 1.

### Running time

It should be noted that the process of identifying conserved genetic markers between draft and reference chromosomes dominates the overall running time of CAR. For example, in the experiments performed above, the average running time of CAR for a pair of draft and reference chromosomes is 15.96 seconds when running with NUCmer and 86.51 seconds with PROmer. In the former case, however, NUCmer takes about 14.56 seconds and in the latter case, PROmer takes about 76.06 seconds. Considering both cases, CAR itself takes on average between 1.40 and 10.45 seconds to finish the assembly of contigs.

## Conclusions

Contig assembly (scaffolding) is a process of ordering and orienting contigs of a draft genome, which is important and helpful to the finishing of a genome sequencing project. In this study, we introduced CAR, an easy-to-use contig assembly tool, that can efficiently produce a more accurate contig assembly of a prokaryotic draft genome based on a reference genome of a related organism. CAR was implemented based on a linear time algorithm we recently developed using genome rearrangements and permutation groups in algebra. For the size of prokaryotic chromosomes, CAR was able to finish its contig assembly job in several seconds to a couple of minutes. When compared to other tools using a real dataset composed of several prokaryotic genomes, CAR exhibited the best performance in sensitivity, precision and genome coverage in reference-based contig assembly.

## Availability and requirements

**Project name:** CAR **Project home page:**http://genome.cs.nthu.edu.tw/CAR/**Operating system(s):** Linux **Programming language:** PHP **Other requirements:** MUMmer **License:** GNU GPL **Any restrictions to use by non-academics:** None

## Additional file

Additional file 1
**Performance results on individual query genomes.** **Table S1:** Performance of sensitivity on individual query chromosomes using closest reference chromosomes. **Table S2:** Performance of average sensitivity on individual query chromosomes using top 10 closest reference chromosomes. **Table S3:** Performance of average sensitivity on individual query chromosomes using top 20 closest reference chromosomes. **Table S4:** Performance of precision on individual query chromosomes using closest reference chromosomes. **Table S5:** Performance of average precision on individual query chromosomes using top 10 closest reference chromosomes. **Table S6:** Performance of average precision on individual query chromosomes using top 20 closest reference chromosomes. **Table S7:** Performance of genome coverage on individual query chromosomes using closest reference chromosomes. **Table S8:** Performance of average genome coverage on individual query chromosomes using top 10 closest reference chromosomes. **Table S9:** Performance of average genome coverage on individual query chromosomes using top 20 closest reference chromosomes.
