# Supplementary material for: CAR: contig assembly of prokaryotic draft genomes using rearrangements
Source: BMC Bioinformatics. 2014 Nov 28;15(1):381. doi: 10.1186/s12859-014-0381-3 (PMC4253983; doi:10.1186/s12859-014-0381-3)
Supplement: Additional file 1 — Performance results on individual query genomes. Table S1: Performance of sensitivity on individual query chromosomes using closest reference chromosomes. Table S2: Performance of average sensitivity on individual query chromosomes using top 10 closest reference chromosomes. Table S3: Performance of average sensitivity on individual query chromosomes using top 20 closest reference chromosomes. Table S4: Performance of precision on individual query chromosomes using closest reference chromosomes. Table S5: Performance of average precision on individual query chromosomes using top 10 closest reference chromosomes. Table S6: Performance of average precision on individual query chromosomes using top 20 closest reference chromosomes. Table S7: Performance of genome coverage on individual query chromosomes using closest reference chromosomes. Table S8: Performance of average genome coverage on individual query chromosomes using top 10 closest reference chromosomes. Table S9: Performance of average genome coverage on individual query chromosomes using top 20 closest reference chromosomes. [file 12859_2014_381_MOESM1_ESM.pdf]

Performance of sensitivity on individual query genomes

Table S1 shows the sensitivity performances obtained by all contig assembly tools on individual query chromosomes when using the closest chromosome as the reference. As a result, most tools perform well on the query chromosomes with a small number of contigs, but not on the ones with a high number of contigs. Moreover, according to the counts in the "Total" row of Table S1, the sensitivity performance of CAR (running with PROmer) is the best among all contig assembly tools. Tables S2 and S3 further show the performance results of all tools on average sensitivity when the top 10 and top 20 closest chromosomes, respectively, are used as the references. Again, the number of counts that CAR (PROmer) gives the best average sensitivity is the highest.

Performance of precision on individual query genomes

Table S4 presents the precision performances of all contig assembly tools applied on individual query chromosomes when the closest chromosome is used as the reference. As shown in this table, most tools have well performance when the number of contigs in a query chromosome is low. However, their performances are reduced when the number of contigs becomes higher. Overall speaking, CAR (PROmer) exhibits the best performance when considering the total number of counts that a tool gives the best precision. As further shown in Table S5, when using the top 10 closest chromosomes as the references, OSLay has the best performance and CAR (PROmer) acts as the second best in terms of the average precision. However, if the the top 20 closest chromosomes are used as the references, then CAR (PROmer) still holds the best on the performance of average precision as revealed in Table S6.

Performance of genome coverage on individual query genomes

Tables S7–S9 demonstrate the genome coverage performances of all contig assembly tools applied on individual query chromosomes when the references are the top 1, top 10 and top 20 closest chromosomes, respectively. As shown in Table S7, the performances of genome coverage for most tools are well on the chromosomes with a small number of contigs, when using the closest chromosome as the reference. In this case, CAR (PROmer), CAR (NUCmer) and Mauve Aligner are all the best tools based on the total counts shown in the last row of Table S7. However, when the top 10 or 20 closest chromosomes are instead used as the references, only CAR (PROmer) gives the best performance in terms of average genome coverage as shown in Tables S8 and S9, respectively.

Table S1: Performance of sensitivity on individual query chromosomes using closest reference chromosomes.

Query chromosome	#Contig	CAR (PROmer)	SIS (PROmer)	Mauve Aligner	r2cat	CAR (NUCmer)	SIS (NUCmer)	OSLay	fillScaffolds (NUCmer)	fillScaffolds (PROmer)	CONTIGuator	Projector2	ABACAS
C. muridarum Nigg	4	66.7	66.7	**100.0**	**100.0**	**100.0**	**100.0**	**100.0**	**100.0**	33.3	**100.0**	**100.0**	0.0
B. subtilis 168	5	**100.0**	75.0	75.0	**100.0**	**100.0**	75.0	75.0	50.0	50.0	**100.0**	75.0	50.0
B. melitensis bv 1 16M (II)	12	63.6	54.5	36.4	**81.8**	**81.8**	72.7	**81.8**	72.7	45.5	54.5	36.4	54.5
B. thailandensis E264 (II)	15	**100.0**	**100.0**	**100.0**	**100.0**	**100.0**	**100.0**	**100.0**	**100.0**	85.7	**100.0**	85.7	28.6
Y. pestis Nepal516	17	68.8	68.8	56.3	43.8	**81.3**	**81.3**	62.5	31.3	12.5	43.8	25.0	37.5
M. genitalium G37	24	**73.9**	**73.9**	65.2	56.5	0.0	0.0	0.0	0.0	60.9	0.0	39.1	0.0
B. thailandensis E264 (I)	28	**92.6**	**92.6**	**92.6**	88.9	**92.6**	**92.6**	**92.6**	**92.6**	77.8	85.2	81.5	51.9
Vibrio Ex25 (II)	33	**90.6**	**90.6**	78.1	78.1	71.9	71.9	65.6	59.4	65.6	50.0	43.8	15.6
B. pinnipedialis B2 94 (II)	34	57.6	57.6	57.6	57.6	**60.6**	**60.6**	**60.6**	**60.6**	57.6	57.6	57.6	**60.6**
A. boonei T469	35	**17.6**	14.7	5.9	0.0	0.0	0.0	0.0	0.0	2.9	0.0	0.0	0.0
B. melitensis bv 1 16M (I)	41	67.5	67.5	77.5	85.0	75.0	75.0	70.0	72.5	67.5	72.5	55.0	**87.5**
S. sputigena ATCC 35185	53	**3.8**	1.9	0.0	0.0	0.0	0.0	0.0	0.0	0.0	0.0	0.0	0.0
B. pinnipedialis B2 94 (I)	55	87.0	87.0	92.6	88.9	**94.4**	**94.4**	**94.4**	**94.4**	87.0	81.5	81.5	88.9
B. longum DJO10A	58	71.9	71.9	77.2	**78.9**	73.7	73.7	54.4	56.1	50.9	47.4	47.4	43.9
C. aurimucosum ATCC 700975	90	**44.9**	42.7	36.0	43.8	18.0	18.0	1.1	14.6	11.2	1.1	23.6	0.0
C. efficiens YS 314	118	**64.1**	**64.1**	**64.1**	59.0	32.5	32.5	5.1	27.4	45.3	19.7	40.2	0.0
M. luteus NCTC 2665	126	**44.8**	**44.8**	35.2	34.4	21.6	20.8	4.8	13.6	16.0	7.2	12.0	0.8
Vibrio Ex25 (I)	176	92.6	92.0	**96.0**	92.0	81.7	81.1	65.1	78.9	90.3	65.1	30.3	64.6
S. pneumoniae TIGR4	209	78.8	78.8	**88.0**	83.2	84.6	84.1	85.6	76.0	74.0	70.2	61.5	84.6
M. tuberculosis H37Ra	220	84.5	84.5	89.0	90.0	**98.2**	**98.2**	74.0	97.7	79.0	65.3	59.4	96.3
S. erythraea NRRL 2338	238	**19.0**	18.6	16.9	13.9	16.0	15.2	6.3	12.2	7.2	10.1	11.8	0.4
C. cellulovorans 743B	297	**9.1**	8.8	4.1	4.4	1.0	1.0	0.0	1.0	2.4	1.0	0.0	0.0
S. aurantiaca DW4 3 1	472	**42.7**	41.8	40.8	37.6	27.0	26.3	13.8	17.0	16.8	18.0	12.7	3.0
Total		13	6	6	5	9	7	6	5	0	3	1	2

The column "#Contig" shows the number of contigs used for the experiment in a query chromosome. For every query chromosome, the best results are displayed in bold. The value in the "Total" row indicates the number of counts that the corresponding tool gives the best performance in sensitivity.

Table S2: Performance of average sensitivity on individual query chromosomes using top 10 closest reference chromosomes.

Query chromosome	#Contig	CAR (PROmer)	SIS (PROmer)	Mauve Aligner	r2cat	CAR (NUCmer)	SIS (NUCmer)	OSLay	fillScaffolds (NUCmer)	fillScaffolds (PROmer)	CONTIGuator	Projector2	ABACAS
C. muridarum Nigg	4	63.3	63.3	**76.7**	63.3	63.3	63.3	46.7	40.0	26.7	46.7	40.0	0.0
B. subtilis 168	5	**80.0**	77.5	77.5	72.5	75.0	72.5	15.0	65.0	70.0	47.5	77.5	5.0
B. melitensis bv 1 16M (II)	12	58.2	50.0	43.6	61.8	**71.8**	62.7	61.8	53.6	38.2	49.1	31.8	51.8
B. thailandensis E264 (II)	15	75.0	71.4	67.9	64.3	**82.1**	**82.1**	75.0	43.6	35.7	65.0	52.1	12.9
Y. pestis Nepal516	17	55.0	54.4	41.9	35.0	**65.6**	65.0	65.0	30.0	23.1	33.8	21.9	23.1
M. genitalium G37	24	9.1	8.7	**12.2**	5.7	0.0	0.0	0.0	0.0	7.8	0.0	3.9	0.0
B. thailandensis E264 (I)	28	83.7	82.6	73.3	67.0	**85.9**	**85.9**	84.4	59.6	49.3	68.9	57.0	32.6
Vibrio Ex25 (II)	33	**44.4**	41.3	32.5	24.1	17.5	17.5	9.1	12.2	17.2	9.1	10.6	1.6
B. pinnipedialis B2 94 (II)	34	**60.6**	60.3	60.0	54.2	58.8	58.5	57.6	55.8	57.0	57.3	54.8	57.6
A. boonei T469	35	**15.3**	12.6	3.5	0.9	0.6	0.6	0.0	0.6	1.2	0.0	0.0	0.0
B. melitensis bv 1 16M (I)	41	72.8	71.8	76.0	**79.0**	78.5	78.5	73.5	76.3	67.8	68.5	55.0	78.0
S. sputigena ATCC 35185	53	**1.9**	1.5	0.2	0.4	0.4	0.4	0.0	0.4	1.0	0.0	0.0	0.0
B. pinnipedialis B2 94 (I)	55	85.7	85.7	91.3	88.7	**94.4**	**94.4**	94.3	**94.4**	85.6	80.2	74.8	88.9
B. longum DJO10A	58	58.9	57.4	**59.3**	54.4	53.0	52.5	38.8	36.0	36.5	33.0	26.7	26.8
C. aurimucosum ATCC 700975	90	**34.0**	32.9	30.8	31.8	8.1	8.0	0.4	6.6	8.8	0.1	12.5	0.0
C. efficiens YS 314	118	**46.5**	45.8	42.1	37.6	17.4	17.1	1.9	15.4	28.0	5.9	19.4	0.0
M. luteus NCTC 2665	126	**37.0**	35.9	23.6	22.6	10.9	10.4	2.2	6.9	14.0	3.3	4.9	0.1
Vibrio Ex25 (I)	176	**83.7**	83.2	69.3	59.0	37.1	36.5	19.3	31.0	54.1	26.1	17.6	14.5
S. pneumoniae TIGR4	209	77.8	77.5	**88.1**	83.4	86.1	85.9	85.8	78.8	70.0	72.5	57.8	81.0
M. tuberculosis H37Ra	220	74.0	73.7	77.0	76.2	**78.5**	78.3	58.1	74.3	63.4	54.2	46.5	69.2
S. erythraea NRRL 2338	238	**11.8**	11.4	8.7	8.6	8.7	8.4	1.4	4.0	2.2	1.8	4.2	0.2
C. cellulovorans 743B	297	**8.8**	8.1	5.5	5.6	0.7	0.6	0.3	0.7	2.6	0.5	0.6	0.3
S. aurantiaca DW4 3 1	472	**9.4**	9.1	6.1	5.6	3.2	3.1	1.6	2.1	3.2	1.9	1.5	0.3
Total		12	0	4	1	6	3	0	1	0	0	0	0

The column "#Contig" shows the number of contigs used for the experiment in a query chromosome. The sensitivity obtained by each tool is averaged on all instances when using the top 10 closest chromosomes as the references. For every query chromosome, the best results are displayed in bold. The value in the "Total" row indicates the number of counts that the corresponding tool gives the best performance in terms of average sensitivity.

Table S3: Performance of average sensitivity on individual query chromosomes using top 20 closest reference chromosomes.

Query chromosome	#Contig	CAR (PROmer)	SIS (PROmer)	Mauve Aligner	r2cat	CAR (NUCmer)	SIS (NUCmer)	OSLay	fillScaffolds (NUCmer)	fillScaffolds (PROmer)	CONTIGuator	Projector2	ABACAS
C. muridarum Nigg	4	45.0	45.0	**55.0**	40.0	36.7	36.7	23.3	26.7	20.0	23.3	25.0	0.0
B. subtilis 168	5	46.3	43.8	42.5	43.8	37.5	36.3	7.5	35.0	46.3	23.8	**53.8**	2.5
B. melitensis bv 1 16M (II)	12	31.4	26.8	22.7	31.4	**37.7**	31.8	31.4	28.6	20.0	25.0	17.3	25.9
B. thailandensis E264 (II)	15	66.8	63.6	57.9	51.8	**68.6**	66.4	56.1	29.3	25.0	42.1	37.1	7.9
Y. pestis Nepal516	17	**48.4**	47.5	34.4	28.8	47.5	46.6	37.2	19.1	19.1	21.9	15.3	12.8
M. genitalium G37	24	5.4	4.8	**8.3**	2.8	0.0	0.0	0.0	0.0	4.8	0.0	2.0	0.0
B. thailandensis E264 (I)	28	83.3	81.9	79.4	70.4	**86.5**	**86.5**	81.7	64.4	48.7	71.1	54.4	23.0
Vibrio Ex25 (II)	33	**27.8**	25.2	18.8	13.1	9.5	9.5	4.5	6.6	10.2	4.5	5.5	0.8
B. pinnipedialis B2 94 (II)	34	**32.7**	32.0	32.1	30.0	31.2	31.1	29.7	28.5	28.9	29.2	28.2	29.7
A. boonei T469	35	**13.2**	11.2	2.6	0.9	0.3	0.3	0.0	0.3	1.3	0.0	0.0	0.0
B. melitensis bv 1 16M (I)	41	**52.3**	50.5	47.9	46.9	45.4	44.8	38.8	41.5	36.5	36.1	29.6	39.6
S. sputigena ATCC 35185	53	**2.3**	1.8	0.5	0.6	0.2	0.2	0.0	0.2	0.8	0.0	0.0	0.0
B. pinnipedialis B2 94 (I)	55	**63.6**	63.5	57.3	57.7	54.6	53.6	51.5	51.9	47.9	45.0	41.2	45.3
B. longum DJO10A	58	**39.3**	37.7	37.6	32.9	30.4	30.1	20.2	20.3	22.5	17.0	15.1	13.4
C. aurimucosum ATCC 700975	90	**27.7**	26.7	23.8	23.4	4.9	4.8	0.2	4.0	6.0	0.1	7.1	0.0
C. efficiens YS 314	118	**36.7**	36.0	32.6	29.1	10.6	10.3	1.0	9.6	18.5	2.9	11.6	0.0
M. luteus NCTC 2665	126	**35.7**	34.9	20.0	19.8	8.7	8.2	1.5	5.4	12.0	2.3	4.0	0.1
Vibrio Ex25 (I)	176	**58.8**	58.3	44.6	36.1	19.8	19.4	9.9	16.3	33.5	13.3	9.3	7.5
S. pneumoniae TIGR4	209	56.4	56.0	**61.3**	57.8	59.1	58.8	57.9	53.5	47.5	49.4	38.6	54.3
M. tuberculosis H37Ra	220	**60.5**	60.2	59.4	57.1	50.5	50.2	33.1	46.1	45.0	34.2	28.8	36.1
S. erythraea NRRL 2338	238	**10.7**	10.3	7.9	7.6	6.7	6.3	0.8	2.6	1.9	1.0	3.3	0.1
C. cellulovorans 743B	297	**8.9**	8.3	5.7	5.3	0.5	0.5	0.2	0.5	2.6	0.4	0.6	0.2
S. aurantiaca DW4 3 1	472	**5.6**	5.4	3.5	3.0	1.6	1.6	0.8	1.1	2.0	1.0	0.7	0.1
Total		16	0	3	0	3	1	0	0	0	0	1	0

The column "#Contig" shows the number of contigs used for the experiment in a query chromosome. The sensitivity obtained by each tool is averaged on all instances when using the top 20 closest chromosomes as the references. For every query chromosome, the best results are displayed in bold. The value in the "Total" row indicates the number of counts that the corresponding tool gives the best performance in terms of average sensitivity.

Table S4: Performance of precision on individual query chromosomes using closest reference chromosomes.

Query chromosome	#Contig	CAR (PROmer)	SIS (PROmer)	Mauve Aligner	r2cat	CAR (NUCmer)	SIS (NUCmer)	OSLay	fillScaffolds (NUCmer)	fillScaffolds (PROmer)	CONTIGuator	Projector2	ABACAS
C. muridarum Nigg	4	66.7	66.7	**100.0**	**100.0**	**100.0**	**100.0**	**100.0**	**100.0**	33.3	**100.0**	**100.0**	0.0
B. subtilis 168	5	**100.0**	75.0	75.0	**100.0**	**100.0**	75.0	75.0	50.0	50.0	**100.0**	75.0	**100.0**
B. melitensis bv 1 16M (II)	12	63.6	54.5	36.4	**81.8**	**81.8**	72.7	**81.8**	72.7	45.5	66.7	50.0	54.5
B. thailandensis E264 (II)	15	**100.0**	**100.0**	**100.0**	**100.0**	**100.0**	**100.0**	**100.0**	**100.0**	85.7	**100.0**	92.3	50.0
Y. pestis Nepal516	17	73.3	73.3	56.3	43.8	**81.3**	**81.3**	66.7	31.3	13.3	46.7	33.3	46.2
M. genitalium G37	24	**73.9**	**73.9**	65.2	68.4	0.0	0.0	0.0	0.0	60.9	0.0	64.3	0.0
B. thailandensis E264 (I)	28	**92.6**	**92.6**	**92.6**	88.9	**92.6**	**92.6**	**92.6**	**92.6**	77.8	88.5	88.0	73.7
Vibrio Ex25 (II)	33	**93.5**	**93.5**	78.1	80.6	82.1	82.1	80.8	67.9	67.7	64.0	66.7	50.0
B. pinnipedialis B2 94 (II)	34	86.4	86.4	57.6	79.2	**87.0**	**87.0**	**87.0**	**87.0**	86.4	86.4	86.4	**87.0**
A. boonei T469	35	**24.0**	20.0	5.9	0.0	0.0	0.0	0.0	0.0	4.0	0.0	0.0	0.0
B. melitensis bv 1 16M (I)	41	77.1	77.1	77.5	85.0	76.9	76.9	73.7	74.4	77.1	85.3	71.0	**89.7**
S. sputigena ATCC 35185	53	**5.6**	2.8	0.0	0.0	0.0	0.0	0.0	0.0	0.0	0.0	0.0	0.0
B. pinnipedialis B2 94 (I)	55	90.4	90.4	92.6	88.9	**94.4**	**94.4**	**94.4**	**94.4**	90.4	89.8	89.8	88.9
B. longum DJO10A	58	78.8	78.8	77.2	**78.9**	77.8	77.8	75.6	59.3	55.8	73.0	77.1	61.0
C. aurimucosum ATCC 700975	90	58.8	55.9	36.0	**60.0**	36.4	36.4	50.0	29.5	14.7	12.5	47.7	0.0
C. efficiens YS 314	118	**72.8**	**72.8**	64.1	68.3	53.5	53.5	42.9	45.1	51.5	47.9	61.8	0.0
M. luteus NCTC 2665	126	**52.8**	**52.8**	35.2	40.6	39.7	38.2	42.9	25.0	18.9	23.7	41.7	50.0
Vibrio Ex25 (I)	176	93.1	92.5	**96.0**	92.0	87.7	87.1	76.0	84.7	90.8	78.6	56.4	79.6
S. pneumoniae TIGR4	209	83.2	83.2	88.0	84.4	88.0	87.5	**91.3**	79.0	78.2	83.4	77.6	90.7
M. tuberculosis H37Ra	220	90.2	90.2	89.0	90.0	**98.6**	**98.6**	86.6	98.2	84.4	81.7	79.3	97.7
S. erythraea NRRL 2338	238	30.0	29.3	16.9	21.4	35.2	33.3	**55.6**	26.9	11.3	42.1	36.8	33.3
C. cellulovorans 743B	297	17.0	16.4	4.1	10.3	7.9	7.9	0.0	7.9	4.4	**42.9**	0.0	0.0
S. aurantiaca DW4 3 1	472	**51.5**	50.5	40.8	46.0	44.4	43.4	50.0	28.0	20.3	42.7	35.3	25.9
Total		10	6	4	6	9	7	8	5	0	4	1	3

The column "#Contig" shows the number of contigs used for the experiment in a query chromosome. For every query chromosome, the best results are displayed in bold. The value in the "Total" row indicates the number of counts that the corresponding tool gives the best performance in precision.

Table S5: Performance of average precision on individual query chromosomes using top 10 closest reference chromosomes.

Query chromosome	#Contig	CAR (PROmer)	SIS (PROmer)	Mauve Aligner	r2cat	CAR (NUCmer)	SIS (NUCmer)	OSLay	fillScaffolds (NUCmer)	fillScaffolds (PROmer)	CONTIGuator	Projector2	ABACAS
C. muridarum Nigg	4	63.3	63.3	**76.7**	63.3	63.3	63.3	46.7	40.0	26.7	46.7	41.7	0.0
B. subtilis 168	5	80.0	77.5	77.5	72.5	79.2	76.7	17.5	66.7	70.0	47.5	**80.8**	10.0
B. melitensis bv 1 16M (II)	12	64.9	55.9	43.6	63.9	**76.7**	66.7	70.0	56.2	42.2	64.5	45.4	58.9
B. thailandensis E264 (II)	15	81.0	76.8	67.9	66.2	**89.3**	**89.3**	85.3	45.8	37.4	72.2	59.7	28.6
Y. pestis Nepal516	17	60.7	59.9	41.9	35.0	65.8	65.2	**69.3**	30.0	25.6	36.0	25.3	35.7
M. genitalium G37	24	9.8	9.3	**12.2**	6.8	0.0	0.0	0.0	0.0	8.9	0.0	6.4	0.0
B. thailandensis E264 (I)	28	85.2	84.1	73.3	67.0	87.4	87.4	**88.2**	60.1	49.7	72.1	62.7	50.3
Vibrio Ex25 (II)	33	**47.3**	43.9	32.5	28.3	24.9	24.8	14.7	16.5	18.0	11.8	22.8	5.0
B. pinnipedialis B2 94 (II)	34	**87.9**	87.5	60.0	73.3	83.4	83.0	83.8	78.9	82.7	86.7	84.5	83.5
A. boonei T469	35	**20.9**	17.3	3.5	10.3	10.0	10.0	0.0	10.0	1.6	0.0	0.0	0.0
B. melitensis bv 1 16M (I)	41	80.6	79.5	76.0	80.3	**83.1**	**83.1**	81.1	80.5	74.9	82.1	71.1	82.3
S. sputigena ATCC 35185	53	**9.9**	9.3	0.2	1.1	8.3	8.3	0.0	8.3	8.6	0.0	0.0	0.0
B. pinnipedialis B2 94 (I)	55	91.7	91.7	91.3	89.6	95.6	95.6	**96.1**	95.6	91.5	89.8	85.4	91.2
B. longum DJO10A	58	66.3	64.6	59.3	58.3	61.4	60.9	**67.3**	39.7	40.9	55.4	48.4	42.2
C. aurimucosum ATCC 700975	90	45.1	43.6	30.8	**45.7**	22.7	22.3	20.0	19.1	11.6	1.3	33.8	0.0
C. efficiens YS 314	118	**57.8**	56.9	42.4	52.3	38.4	37.6	39.8	33.7	34.5	20.2	44.4	0.0
M. luteus NCTC 2665	126	**44.4**	43.2	23.6	28.9	25.0	23.9	31.1	15.3	16.5	18.0	19.5	5.0
Vibrio Ex25 (I)	176	**85.4**	84.9	69.3	64.4	53.3	52.1	53.0	43.2	55.1	43.0	39.0	31.1
S. pneumoniae TIGR4	209	82.5	82.2	88.1	84.6	88.6	88.4	**91.0**	81.1	74.3	85.0	75.0	88.6
M. tuberculosis H37Ra	220	82.0	81.7	77.0	81.0	**86.2**	85.9	77.1	80.9	69.6	72.4	68.4	78.2
S. erythraea NRRL 2338	238	20.0	19.3	8.7	14.8	23.5	22.5	**24.5**	10.5	3.6	16.9	19.2	20.0
C. cellulovorans 743B	297	18.3	16.9	5.5	15.4	9.3	7.5	**46.7**	9.3	5.3	26.6	16.3	12.2
S. aurantiaca DW4 3 1	472	15.3	14.9	6.1	9.6	8.6	8.1	**36.9**	6.3	5.1	13.8	7.8	2.6
Total		7	0	2	1	4	2	8	0	0	0	1	0

The column "#Contig" shows the number of contigs used for the experiment in a query chromosome. The precision obtained by each tool is averaged on all instances when using the top 10 closest chromosomes as the references. For every query chromosome, the best results are displayed in bold. The value in the "Total" row indicates the number of counts that the corresponding tool gives the best performance in terms of average precision.

Table S6: Performance of average precision on individual query chromosomes using top 20 closest reference chromosomes.

Query chromosome	#Contig	CAR (PROmer)	SIS (PROmer)	Mauve Aligner	r2cat	CAR (NUCmer)	SIS (NUCmer)	OSLay	fillScaffolds (NUCmer)	fillScaffolds (PROmer)	CONTIGuator	Projector2	ABACAS
C. muridarum Nigg	4	45.0	45.0	**55.0**	40.0	36.7	36.7	23.3	26.7	20.0	23.3	26.7	0.0
B. subtilis 168	5	46.3	43.8	42.5	43.8	39.6	38.3	8.8	38.3	46.3	23.8	**64.6**	5.0
B. melitensis bv 1 16M (II)	12	35.8	30.6	22.7	32.8	**43.0**	35.0	40.0	31.8	22.4	37.3	26.3	29.4
B. thailandensis E264 (II)	15	73.6	69.8	57.9	55.3	**76.7**	74.2	74.6	31.7	26.8	53.7	45.3	34.3
Y. pestis Nepal516	17	**52.1**	51.1	34.4	29.1	50.3	49.0	39.9	20.1	20.6	25.8	18.5	19.8
M. genitalium G37	24	6.4	5.4	**8.3**	3.4	0.0	0.0	0.0	0.0	6.2	0.0	3.2	0.0
B. thailandensis E264 (I)	28	84.4	82.9	79.4	70.5	**88.0**	**88.0**	86.2	65.3	49.1	76.8	61.8	41.2
Vibrio Ex25 (II)	33	**30.2**	27.2	18.8	15.9	15.9	15.8	7.4	10.4	10.8	5.9	12.7	2.5
B. pinnipedialis B2 94 (II)	34	48.0	46.7	32.1	41.6	46.8	46.6	**59.9**	41.8	42.1	49.7	46.0	58.9
A. boonei T469	35	**18.3**	15.5	2.6	9.8	5.0	5.0	0.0	5.0	1.8	0.0	0.0	0.0
B. melitensis bv 1 16M (I)	41	59.5	57.4	47.9	49.8	51.5	50.4	**86.4**	45.8	40.7	55.7	41.7	44.7
S. sputigena ATCC 35185	53	**9.6**	8.8	0.5	5.3	4.2	4.2	0.0	4.2	7.1	0.0	0.0	0.0
B. pinnipedialis B2 94 (I)	55	70.6	70.5	57.3	61.4	60.0	57.9	**88.6**	54.8	51.8	64.6	50.7	50.1
B. longum DJO10A	58	**47.1**	45.1	37.6	38.7	39.7	39.4	40.1	25.9	26.3	31.5	30.8	21.1
C. aurimucosum ATCC 700975	90	**37.2**	35.9	23.8	34.7	15.5	15.3	10.0	12.9	8.0	0.6	23.5	0.0
C. efficiens YS 314	118	**47.4**	46.5	32.8	43.0	28.1	27.1	27.4	25.7	23.4	10.1	34.4	0.0
M. luteus NCTC 2665	126	43.5	42.4	20.0	26.3	22.7	21.3	**47.0**	14.2	14.4	14.8	19.0	5.0
Vibrio Ex25 (I)	176	**61.5**	61.1	44.6	41.0	30.7	29.8	42.7	24.2	34.6	23.1	22.7	18.1
S. pneumoniae TIGR4	209	60.6	60.1	61.3	59.2	61.2	60.8	**62.1**	55.2	50.4	58.2	50.8	60.1
M. tuberculosis H37Ra	220	**72.0**	71.6	59.6	67.6	66.9	66.4	64.9	59.2	52.3	54.0	51.8	54.1
S. erythraea NRRL 2338	238	18.5	17.7	7.9	13.2	**19.0**	18.0	18.3	7.1	3.2	12.2	17.0	15.0
C. cellulovorans 743B	297	18.3	17.2	5.7	14.9	6.8	5.9	**28.3**	6.8	5.4	21.3	16.8	9.1
S. aurantiaca DW4 3 1	472	10.2	9.8	3.5	5.7	5.3	5.1	**18.4**	4.2	3.6	6.9	3.9	1.3
Total		9	0	2	0	4	1	7	0	0	0	1	0

The column "#Contig" shows the number of contigs used for the experiment in a query chromosome. The precision obtained by each tool is averaged on all instances when using the top 20 closest chromosomes as the references. For every query chromosome, the best results are displayed in bold. The value in the "Total" row indicates the number of counts that the corresponding tool gives the best performance in terms of average precision.

Table S7: Performance of genome coverage on individual query chromosomes using closest reference chromosomes.

Query chromosome	#Contig	CAR (PROmer)	SIS (PROmer)	Mauve Aligner	r2cat	CAR (NUCmer)	SIS (NUCmer)	OSLay	fillScaffolds (NUCmer)	fillScaffolds (PROmer)	CONTIGuator	Projector2	ABACAS
C. muridarum Nigg	4	79.8	50.0	**86.8**	**86.8**	**86.8**	**86.8**	**86.8**	**86.8**	42.9	**86.8**	**86.8**	0.0
B. subtilis 168	5	**60.1**	56.6	56.6	**60.1**	**60.1**	56.6	55.4	10.8	10.8	**60.1**	15.5	8.2
B. melitensis bv 1 16M (II)	12	58.7	49.3	41.1	**76.1**	**76.1**	58.8	**76.1**	68.1	41.3	66.4	46.0	53.6
B. thailandensis E264 (II)	15	**91.3**	**91.3**	**91.3**	**91.3**	**91.3**	**91.3**	**91.3**	**91.3**	76.9	**91.3**	80.5	10.1
Y. pestis Nepal516	17	73.8	73.8	43.2	18.9	**76.1**	**76.1**	68.1	26.6	18.0	32.5	34.7	12.6
M. genitalium G37	24	61.1	61.1	**67.2**	62.3	0.0	0.0	0.0	0.0	42.7	0.0	57.4	0.0
B. thailandensis E264 (I)	28	**74.7**	**74.7**	**74.7**	71.1	**74.7**	**74.7**	**74.7**	**74.7**	55.0	72.9	65.0	14.8
Vibrio Ex25 (II)	33	86.8	**90.6**	72.3	74.6	81.4	85.4	73.6	65.5	51.9	51.6	51.6	4.0
B. pinnipedialis B2 94 (II)	34	83.9	83.9	83.9	75.9	**86.0**	**86.0**	**86.0**	**86.0**	83.9	83.9	83.9	85.8
A. boonei T469	35	**9.7**	5.6	1.0	0.0	0.0	0.0	0.0	0.0	0.5	0.0	0.0	0.0
B. melitensis bv 1 16M (I)	41	84.4	84.4	89.1	90.4	85.3	85.3	76.9	78.9	84.4	85.5	73.8	**95.1**
S. sputigena ATCC 35185	53	**6.3**	1.7	0.0	0.0	0.0	0.0	0.0	0.0	0.0	0.0	0.0	0.0
B. pinnipedialis B2 94 (I)	55	86.6	86.6	91.1	90.4	**95.4**	**95.4**	**95.4**	**95.4**	86.6	85.6	85.6	90.4
B. longum DJO10A	58	77.0	77.0	**86.1**	81.8	77.3	76.8	66.8	61.5	60.1	72.4	74.7	29.0
C. aurimucosum ATCC 700975	90	**56.5**	53.5	42.4	54.5	28.9	28.9	2.4	22.1	8.4	1.5	35.4	0.0
C. efficiens YS 314	118	76.9	77.3	**77.8**	71.7	50.8	50.8	7.1	39.2	56.8	25.2	55.9	0.0
M. luteus NCTC 2665	126	**49.3**	**49.3**	37.0	34.7	27.1	25.8	5.2	15.1	14.8	11.0	19.2	0.1
Vibrio Ex25 (I)	176	91.4	90.7	**95.8**	82.4	86.0	85.5	71.4	79.6	83.4	70.1	49.5	45.7
S. pneumoniae TIGR4	209	80.2	80.5	**90.0**	86.6	87.1	86.9	89.2	78.0	76.2	77.3	68.1	85.2
M. tuberculosis H37Ra	220	89.7	89.7	91.9	88.0	**98.2**	**98.2**	81.2	98.0	84.0	78.0	73.9	96.6
S. erythraea NRRL 2338	238	**29.8**	**29.8**	26.3	20.3	26.4	24.9	12.1	17.9	8.1	14.7	21.7	0.0
C. cellulovorans 743B	297	**15.8**	14.8	4.6	5.7	2.3	2.3	0.0	2.3	4.1	1.1	0.0	0.0
S. aurantiaca DW4 3 1	472	42.1	42.3	**42.8**	38.3	33.7	33.1	15.6	17.1	11.7	20.7	14.1	0.9
Total		9	5	9	4	9	7	6	5	0	3	1	1

The column "#Contig" shows the number of contigs used for the experiment in a query chromosome. For every query chromosome, the best results are displayed in bold. The value in the "Total" row indicates the number of counts that the corresponding tool gives the best performance in genome coverage.

Table S8: Performance of average genome coverage on individual query chromosomes using top 10 closest reference chromosomes.

Query chromosome	#Contig	CAR (PROmer)	SIS (PROmer)	Mauve Aligner	r2cat	CAR (NUCmer)	SIS (NUCmer)	OSLay	fillScaffolds (NUCmer)	fillScaffolds (PROmer)	CONTIGuator	Projector2	ABACAS
C. muridarum Nigg	4	55.2	57.0	**64.0**	50.4	52.2	63.0	49.3	25.2	15.8	37.4	25.2	0.0
B. subtilis 168	5	44.3	**47.7**	**47.7**	42.4	47.0	46.6	11.1	36.9	38.1	29.3	47.3	0.8
B. melitensis bv 1 16M (II)	12	53.7	47.0	47.0	52.9	**61.4**	52.9	52.7	44.5	35.0	54.3	33.3	45.8
B. thailandensis E264 (II)	15	70.9	65.7	62.8	58.1	77.8	**78.2**	70.4	42.7	34.1	58.4	48.2	4.5
Y. pestis Nepal516	17	59.4	59.1	37.6	23.2	**65.0**	64.4	64.8	21.4	19.3	26.5	18.3	6.0
M. genitalium G37	24	8.0	7.9	**10.9**	6.2	0.0	0.0	0.0	0.0	6.7	0.0	5.7	0.0
B. thailandensis E264 (I)	28	70.3	70.5	55.8	45.8	**72.6**	72.2	68.0	41.4	32.4	49.9	39.0	11.2
Vibrio Ex25 (II)	33	**43.3**	41.4	28.6	23.2	20.7	21.4	9.7	14.4	11.3	8.8	14.2	0.4
B. pinnipedialis B2 94 (II)	34	**85.6**	85.3	85.5	72.0	84.7	84.3	84.7	77.3	76.9	81.4	78.0	78.1
A. boonei T469	35	**11.1**	8.7	3.9	1.3	0.9	0.9	0.0	0.9	1.5	0.0	0.0	0.0
B. melitensis bv 1 16M (I)	41	82.9	81.6	**87.5**	84.3	85.8	85.8	80.3	82.5	77.2	81.4	69.6	83.2
S. sputigena ATCC 35185	53	**2.9**	2.2	0.9	1.2	0.1	0.1	0.0	0.1	1.4	0.0	0.0	0.0
B. pinnipedialis B2 94 (I)	55	87.8	87.8	91.0	89.6	**94.9**	**94.9**	94.7	**94.9**	87.8	85.1	78.2	90.3
B. longum DJO10A	58	**67.4**	66.5	62.2	54.6	60.3	60.4	48.9	35.2	34.1	50.0	41.4	15.9
C. aurimucosum ATCC 700975	90	**42.8**	41.6	38.1	41.5	16.0	15.6	1.0	12.8	7.9	0.1	20.6	0.0
C. efficiens YS 314	118	**58.7**	58.0	53.1	50.4	29.0	28.5	2.3	24.5	35.0	7.6	29.9	0.0
M. luteus NCTC 2665	126	**39.5**	38.4	24.8	24.1	14.6	14.0	2.7	8.2	13.3	4.6	8.7	0.0
Vibrio Ex25 (I)	176	**76.8**	**76.8**	59.4	50.0	43.4	42.4	20.8	30.4	39.6	29.8	22.6	7.4
S. pneumoniae TIGR4	209	80.8	80.4	**90.8**	84.8	87.8	87.7	89.5	80.2	73.1	79.7	65.2	81.1
M. tuberculosis H37Ra	220	79.6	79.6	80.7	76.9	**82.9**	82.6	66.0	77.2	66.1	66.5	58.7	68.0
S. erythraea NRRL 2338	238	**20.8**	20.1	13.9	11.7	16.9	16.2	2.6	6.7	3.1	2.5	7.9	0.0
C. cellulovorans 743B	297	**14.6**	13.4	7.8	7.3	1.3	1.0	0.2	1.3	3.1	0.5	0.8	0.1
S. aurantiaca DW4 3 1	472	**9.1**	9.0	6.1	5.5	3.8	3.7	1.8	2.1	2.2	2.2	1.6	0.1
Total		12	2	5	0	5	2	0	1	0	0	0	0

The column "#Contig" shows the number of contigs used for the experiment in a query chromosome. The genome coverage obtained by each tool is averaged on all instances when using the top 10 closest chromosomes as the references. For every query chromosome, the best results are displayed in bold. The value in the "Total" row indicates the number of counts that the corresponding tool gives the best performance in terms of average genome coverage.

Table S9: Performance of average genome coverage on individual query chromosomes using top 20 closest reference chromosomes.

Query chromosome	#Contig	CAR (PROmer)	SIS (PROmer)	Mauve Aligner	r2cat	CAR (NUCmer)	SIS (NUCmer)	OSLay	fillScaffolds (NUCmer)	fillScaffolds (PROmer)	CONTIGuator	Projector2	ABACAS
C. muridarum Nigg	4	37.0	39.4	**49.3**	33.2	30.7	36.1	24.6	15.8	10.8	18.7	15.2	0.0
B. subtilis 168	5	23.5	24.9	26.5	22.6	23.5	23.3	5.5	22.9	29.2	14.6	**42.4**	0.4
B. melitensis bv 1 16M (II)	12	29.0	25.2	24.4	26.9	**32.8**	26.9	26.8	25.1	18.2	27.2	19.2	22.9
B. thailandensis E264 (II)	15	66.4	63.3	56.4	47.6	**68.6**	67.1	57.8	31.6	27.4	34.3	36.5	2.2
Y. pestis Nepal516	17	**49.2**	48.7	28.3	19.3	48.5	47.8	37.4	15.7	16.9	18.3	13.6	3.2
M. genitalium G37	24	4.7	4.2	**6.1**	3.1	0.0	0.0	0.0	0.0	4.6	0.0	2.9	0.0
B. thailandensis E264 (I)	28	67.9	67.7	62.5	49.8	**70.9**	70.7	65.0	44.5	29.6	51.8	37.8	6.3
Vibrio Ex25 (II)	33	**25.6**	23.7	15.8	13.3	11.3	11.7	4.9	7.8	6.4	4.4	7.6	0.2
B. pinnipedialis B2 94 (II)	34	**46.7**	45.9	45.5	40.1	44.9	44.7	43.4	40.1	39.0	41.5	40.5	39.1
A. boonei T469	35	**9.7**	8.1	2.9	1.3	0.4	0.4	0.0	0.4	1.6	0.0	0.0	0.0
B. melitensis bv 1 16M (I)	41	**58.0**	55.9	54.0	51.3	49.5	48.8	41.3	45.2	40.7	41.9	36.7	42.0
S. sputigena ATCC 35185	53	**4.7**	3.6	0.9	0.7	0.1	0.1	0.0	0.1	1.2	0.0	0.0	0.0
B. pinnipedialis B2 94 (I)	55	**66.0**	65.9	57.8	58.7	56.2	54.8	52.1	52.1	49.5	48.5	43.7	45.6
B. longum DJO10A	58	**43.4**	41.9	37.0	34.3	37.1	36.9	25.2	20.7	20.6	25.2	24.4	7.9
C. aurimucosum ATCC 700975	90	**34.1**	32.8	29.7	30.6	9.5	9.4	0.5	7.6	5.2	0.1	12.2	0.0
C. efficiens YS 314	118	**47.5**	46.6	41.6	39.7	17.5	17.1	1.2	15.4	22.8	3.8	19.2	0.0
M. luteus NCTC 2665	126	**38.1**	37.1	21.3	21.2	11.7	10.9	2.0	6.5	11.5	3.0	7.3	0.0
Vibrio Ex25 (I)	176	53.3	**53.5**	36.6	30.4	23.6	22.8	10.5	16.0	23.3	15.1	12.0	3.7
S. pneumoniae TIGR4	209	58.9	58.4	**63.4**	58.7	60.3	60.1	60.5	54.3	49.0	54.3	43.4	54.1
M. tuberculosis H37Ra	220	**67.0**	66.7	64.5	60.0	58.1	58.0	38.3	50.7	45.3	43.9	38.8	34.4
S. erythraea NRRL 2338	238	**19.3**	18.5	12.7	10.4	13.1	12.4	1.5	4.2	2.8	1.3	6.3	0.0
C. cellulovorans 743B	297	**14.5**	13.6	8.0	7.3	0.9	0.7	0.1	0.9	2.9	0.4	0.9	0.1
S. aurantiaca DW4 3 1	472	**5.3**	5.2	3.3	2.9	1.9	1.9	0.9	1.0	1.4	1.1	0.8	0.0
Total		15	1	3	0	3	0	0	0	0	0	1	0

The column "#Contig" shows the number of contigs used for the experiment in a query chromosome. The genome coverage obtained by each tool is averaged on all instances when using the top 20 closest chromosomes as the references. For every query chromosome, the best results are displayed in bold. The value in the "Total" row indicates the number of counts that the corresponding tool gives the best performance in terms of average genome coverage.
